# Minimally Invasive Approaches in Locally Advanced Cervical Cancer Patients Undergoing Radical Surgery After Chemoradiotherapy: A Propensity Score Analysis

**DOI:** 10.1245/s10434-020-09302-y

**Published:** 2020-11-09

**Authors:** G. Ferrandina, V. Gallotta, A. Federico, F. Fanfani, A. Ercoli, V. Chiantera, F. Cosentino, L. C. Turco, F. Legge, L. Pedone Anchora, N. Bizzarri, R. Moroni, G. Macchia, V. Valentini, G. Scambia

**Affiliations:** 1 Gynecologic Oncology Unit, Fondazione Policlinico Universitario A. Gemelli, IRCCS, Rome, Italy; 2 Istituto di Ginecologia e Ostetricia, Università Cattolica del Sacro Cuore, Rome, Italy; 3 Department of Obstetrics and Gynecology, University of Messina, Messina, Italy; 4 Department of Gynecologic Oncology, University of Palermo, Palermo, Italy; 5 Gynecologic Oncology, Gemelli Molise, Campobasso, Italy; 6 Gynecology and Breast Care Unit, Mater Olbia Hospital, Olbia, Italy; 7 Gynecologic Oncology Unit, Department Obstetrics/Gynecology “F. Miulli” General Regional Hospital, Acquaviva delle Fonti, Bari, Italy; 8 Direzione Scientifica, Fondazione Policlinico Universitario A.Gemelli, IRCCS, Rome, Italy; 9 Radiotherapy Unit, Gemelli Molise Hospital, Campobasso, Italy; 10 Dipartimento di Scienze Radiologiche, Radioterapiche ed Ematologiche, Fondazione Policlinico Universitario A. Gemelli, IRCCS, UOC di Radioterapia, Rome, Italy; 11 Istituto di Radiologia, Università Cattolica del Sacro Cuore, Rome, Italy

## Abstract

**Purpose:**

Chemoradiation (CT/RT) followed by radical surgery (RS) may play a role in locally advanced cervical cancer (LACC) patients with suboptimal response to CT/RT or in low-income countries with limited access to radiotherapy. Our aim is to evaluate oncological and surgical outcomes of minimally invasive radical surgery (MI-RS) compared with open radical surgery (O-RS).

**Patients and Methods:**

Data for stage IB2–IVA cervical cancer patients managed by CT/RT and RS were retrospectively analyzed.

**Results:**

Beginning with 686 patients, propensity score matching resulted in 462 cases (231 per group), balanced for FIGO stage, lymph node status, histotype, tumor grade, and clinical response to CT/RT. The 5-year disease-free survival (DFS) was 73.7% in the O-RS patients and 73.0% in the MI-RS patients (HR 1.034, 95% CI 0.708–1.512, *p* = 0.861). The 5-year locoregional recurrence rate was 12.5% (O-RS) versus 15.2% (MI-RS) (HR 1.174, 95% CI 0.656–2.104, *p* = 0.588). The 5-year disease-specific survival (DSS) was 80.4% in O-RS patients and 85.3% in the MI-RS group (HR 0.731, 95% CI 0.438–1.220, *p* = 0.228). Estimated blood loss was lower in the MI-RS group (*p* < 0.001), as was length of hospital stay (*p* < 0.001). Early postoperative complications occurred in 77 patients (33.3%) in the O-RS group versus 88 patients (38.1%) in the MI-RS group (*p* = 0.331). Fifty-six (24.2%) patients experienced late postoperative complications in the O-RS group, versus 61 patients (26.4%) in the MI-RS group (*p* = 0.668).

**Conclusion:**

MI-RS and O-RS are associated with similar rates of recurrence and death in LACC patients managed by surgery after CT/RT. No difference in early or late complications was reported.

**Electronic supplementary material:**

The online version of this article (10.1245/s10434-020-09302-y) contains supplementary material, which is available to authorized users.

Cervical cancer (CC) is the fourth most common malignancy in women, with > 500,000 new diagnoses per year and a mortality rate of approximately 50% worldwide.^1^ Locally advanced CC (stage IB > 4 cm-IVA disease) (LACC) accounts for 30–40% of new diagnoses in developed countries^2^ and around 80–90% in low-/middle-income countries.^3^^,^^4^ Exclusive pelvic ± extended-field chemoradiotherapy and utero-vaginal brachytherapy (E-CT/RT) represents the standard of treatment worldwide, providing 5-year overall survival rates between 60 and 75%, according to stage of disease.^5^ Adoption of radical surgery (RS) as an alternative to vaginal brachytherapy after chemoradiation (CT/RT) has been proposed in recent decades to improve local disease control, and reduce radiation dose and potential toxicity.^6^–^10^ In our phase II ROMA-2 study adopting CT/RT with concomitant boost followed by completion surgery, we achieved 50.5% pathological complete response and only 7% rate of 3-year locoregional failure.^8^

Two prospective, randomized studies have investigated the efficacy of CT/RT plus RS versus E-CT/RT in stage IB2-II CC. The GYNECO-002 trial was prematurely closed due to poor accrual,^11^. while the Mexican trial failed to demonstrate a survival advantage of RS versus vaginal brachytherapy after CT/RT.^12^ Two meta-analyses reported a reduced risk of recurrence in patients managed with CT/RT followed by RS versus E-CT/RT but without improvement of overall survival.^13^^,^^14^ In countries with limited access to radiotherapy facilities or in patients achieving partial response, completion surgery could have a role.^14^^,^^15^ Nonetheless, at our institution, completion surgery has been routinely proposed to all patients deemed suitable for successful surgery, including those with clinical complete response to CT/RT, to reduce radiation dose and vaginal brachytherapy sequelae, considering that complete clinical response would require less radical surgery and the rate of postoperative morbidity would be lower.^6^ Moreover, the availability of data relative to the pathologically pathological would have relevant implications in terms of prognostic characterization and choice of adjuvant treatment.^8^

There have been some concerns about the adverse effects related to this multimodal approach. However, relevant advances have been made in radiotherapy technologies, including image-guided adaptive radiotherapy (IGART), thus improving dose delivery, achieving high local control, and reducing morbidity.^16^ Moreover, several studies on early-stage CC (ECC) have shown that minimally invasive radical surgery (MI-RS) could result in better perioperative and postoperative measures compared with open surgery (O-RS).^17^^,^^18^ These data have also been confirmed in LACC patients managed by MI-RS after neoadjuvant chemotherapy (NACT) or preoperative CT/RT,^19^–^26^ but few data have been reported on clinical outcomes in patients managed with CT/RT followed by MI-RS versus O-RS.^27^ In 2018, the multicenter phase III study LACC trial comparing MI-RS versus O-RS ECC failed to achieve the primary end-point (i.e., noninferiority of MI-RS in terms of 5-year disease-free survival).^28^ Since then, large database studies have reported an association between MI-RS and increased rates of recurrence/death in EEC.^29^ Based on these unexpected results, the NCCN guidelines and the European Society of Gynaecologic Oncology Scientific Committee have recommended that laparotomy should be considered the standard surgical approach in early-stage CC.

In this context, the aim of this study is to evaluate the survival outcome of MI-RS versus O-RS after CT/RT in a large, retrospective series of LACC patients by propensity score analysis. In particular, we planned to test whether adoption of MI-RS in LACC patients managed by completion surgery after CT/RT could be noninferior to O-RS in terms of disease-free survival.

Analysis of peri and postoperative outcomes between the two surgical approaches was also carried out.

## Patients and Methods

After obtaining approval from the Institutional Review Board (DIPUSVSP-26-05-2068), we retrospectively collected data for stage IB > 4 cm-IVA CC patients referred to the Gynecologic Oncology Unit of the Catholic University of Rome and Campobasso, and the Gynecologic Oncology Unit of “F. Miulli” Hospital (Acquaviva delle Fonti) Bari, Italy. The study was performed in accordance with the precepts established by the Helsinki Declaration.

Inclusion criteria were the following: age > 18 years, biopsy-proven cervical carcinoma, and FIGO stage IB > 4 cm-IVA patients managed by preoperative CT/RT. All patients had signed written informed consent agreeing to be submitted to all the procedures described and for their data to be collected. Pretreatment work-up included clinical examination, abdominopelvic MRI, complete blood count, and measurement of liver and renal function, plus cystoscopy and proctoscopy if needed. Preoperative chemoradiation was administered as whole-pelvic irradiation in combination with cisplatin-based regimens (40 mg/m^2^ cisplatin per week or 20 mg/m^2^ 2-h intravenous infusion on days 1–4 and 26–30 of treatment) with or without 5-fluorouracil (1000 mg/m^2^, 24-h continuous intravenous infusion on days 1–4 and 27–30). Slightly different schemes of platinum-based chemotherapy (three versus two cycles), radiotherapy (total dose from 39.6 to 50.4 Gy), or upper border of radiation field (L4–L5 versus L3 vertebra) were used.

Clinical response to CT/RT was assessed within 5/6 weeks from completion of treatment by abdominopelvic MRI, according to RECIST criteria.^30^

### Surgical Procedures

All surgical procedures were attempted in patients achieving clinical response to CT/RT or stable disease. MI-RS was performed by either standard straight laparoscopic instrument or robotic platform (Da Vinci Si or Xi, Intuitive Surgical Inc., Sunnyvale, CA, USA) as described in our previous reports.^22^^,^^24^^,^^25^ Uterine manipulator was not used in any minimally invasive surgical procedures, a pad was placed into the vagina at the colpotomy to preserve the pneumoperitoneum, then the specimen was retrieved through the vagina. The vaginal cuff was sutured through transvaginal or endoscopic approach, according to surgeon preference.

Radical hysterectomy (RH) was classified according to the Piver classification.^31^ RH and pelvic lymphadenectomy were carried out in all patients, while aortic lymphadenectomy was performed in case of persistence of pelvic lymph node involvement at posttreatment imaging, intraoperative assessment of involved pelvic lymph nodes at frozen-section analysis and intraoperative evidence of palpable or indurated or fixed pelvic and/or aortic lymph nodes. Perioperative measures, such as estimated blood loss (EBL), operative time (OT), and length of hospital stay (LOS), were also collected. Postoperative early and long-term complications were defined as any adverse event occurring within or after 30 days from surgery, respectively. Surgical morbidity was classified according to the Chassagne grading system.^32^

### Evaluation of Pathological Response

Residual disease was evaluated based on the examination of uterus, vaginal cuff, parametrium, and pelvic and aortic LNs, and expressed in mm. At histopathological evaluation, the cervix was sectioned clockwise in at least 12 blocks and entirely embedded in paraffin. From each block, 3- to 4-μm-thick slides were cut at different levels and stained with hematoxylin and eosin. Histological evaluation was performed by dedicated pathologists experienced in gynecologic oncology. Pathological response was defined as complete (absence of any residual tumor after treatment at any site level) (pCR), microscopic (persistent tumor foci < 3 mm maximum dimension) (pmicroR), or macroscopic (persistent tumor foci > 3 mm maximum dimension) (pmacroR), according to final pathology.

### Adjuvant Treatment

Patients achieving pathological complete response (pCR) or microscopic partial response (pmicroPR) started routine surveillance procedures, while patients achieving macroscopic partial response (pmacroPR) or involvement of pelvic and/or aortic lymph nodes were triaged to adjuvant chemotherapy.

### Statistical Analysis

The *χ*^2^ test, or Fisher’s exact test for proportion, was used to compare categorical variables. The Mann–Whitney test was used to compare the distribution of continuous variables. Disease-free survival (DFS) was calculated from date of diagnosis to date of relapse or date of last follow-up, and disease-specific survival (DSS) was calculated from date of diagnosis to date of death or date of last follow-up. We used univariate and multivariate Cox proportional hazard models to select the variables with a prognostic role in the whole series. The variables considered in the logistic regression model were chosen on the basis of the preoperative parameters, and their clinical relevance according to the investigators’ opinion. To assess the impact of surgical approach on oncological outcome, we applied the technique of propensity score (PS) matching to reduce bias related to the imbalance of clinical variables between the two treatment groups^33^.

Survival curves were presented as Kaplan–Meier plots.^34^ Cox proportional hazard^35^ models were used to estimate the hazard ratio (HR) and 95% confidence interval (95% CI) for DFS, DSS, as well as the cumulative incidence of locoregional recurrence. Competing-risks models according to the method of Fine and Gray^36^ were used to estimate the hazard ratio and 95% CI for locoregional recurrence, considering distant recurrences as competing risk.

Based on the number of patients in the PS-weighted sample, we calculated the power of the study to declare MI-RS noninferior to O-RS in terms of DFS, considering an expected DFS rate of 75% in patients managed by O-RS in our previous studies,^6^ and a noninferiority margin of 6–8%.

Statistical Package for Social Sciences software version 25.0 (IBM Corporation) and Stata software version 13.0 (StataCorp) were used for statistical analysis.

## Results

**Fig. 1.**
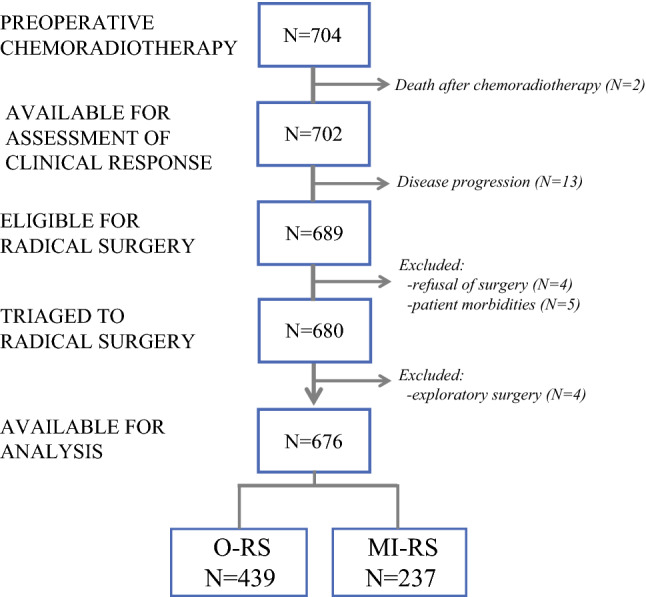
Flowchart of patients

From June 1996 to November 2019, 704 consecutive stage IB2–IVA CC patients underwent preoperative CT/RT. As shown in Fig. 1, two patients died after treatment due to morbidities, leaving 702 patients evaluable for assessment of clinical response. Thirteen patients were deemed as experiencing progression of disease, therefore 689 patients were eligible for completion surgery. After exclusion of 4 cases refusing surgery and 5 cases deemed as unfit for surgery, 680 patients were triaged to RS. Four patients underwent exploratory laparotomy due to presence of abdominal disease, thus leaving 676 patients for analysis (O-RS 439, MI-RS 237). In the MI-RS group, 72 (30.6%) patients were managed by robot-assisted surgery.

The clinicopathological characteristics of patients in the overall series are summarized in Table 1. There was no difference in age or body mass index (BMI) between the two groups. On the other hand, stage IIIB–IVA was more frequently recorded in the O-RS group versus the MI-RS group (14.9% vs. 8.0%, *p* = 0.076). Imbalance between MI-RS and O-RS groups was also documented for lymph node status at imaging, histotype, histological grading, and clinical response to CT/RT (Table 1). To select the variables playing a prognostic role in this clinical setting, we carried out univariate and multivariate analyses of age, FIGO stage, lymph node status, and clinical response to CT/RT. Multivariate analysis of DFS and DSS showed that patients with metastatic aortic lymph nodes ± pelvic lymph nodes, and/or partial response or stable disease after CT/RT were associated with a worse prognosis for both DFS and DSS (Table 2).

**Table 1:** Preoperative clinical and pathological features of the whole population

	All (*N* = 676)	O-RS (*N* = 439)	MI-RS (*N* = 237)	*p* Value^a^
Age, years				
Median (range)	52 (20–83)	53 (20–83)	51 (23–79)	0.179^b^
BMI, kg/m^2^				
Median, range	23.9 (16.4–45)	24.2 (17–42.2)	23.8 (16.4–45)	0.255^b^
FIGO stage				
IB2	57 (8.4)	36 (8.2)	21 (8.9)	
IIA	33 (4.9)	18 (4.1)	15 (6.3)	
IIB	476 (70.4)	303 (69.0)	173 (73.0)	
IIIA	26 (3.8)	17 (3.9)	9 (3.8)	
IIIB	75 (11.1)	56 (12.8)	19 (8.0)	
IVA	9 (1.3)	9 (2.1)	–	0.076^c^
Tumor size				
≤ 4 cm	119 (17.6)	78 (17.8)	41 (17.3)	
> 4 cm	557 (82.4)	361 (82.2)	196 (82.7)	0.916
Lymph node status at imaging				
Negative	402 (59.5)	244 (55.6)	158 (66.7)	
Pelvic positive	253 (37.4)	179 (40.8)	74 (31.2)	
Aortic ± pelvic positive	21 (3.1)	16 (3.6)	5 (2.1)	**0.017**^**c**^
Histotype				
Squamous	596 (88.2)	393 (89.5)	203 (85.7)	
Adenocarcinoma	67 (9.9)	35 (8.0)	32 (13.5)	
Other	13 (1.9)	11 (2.6)	2 (0.8)	**0.027**^**c**^
Grade				
G1–G2	370 (54.8)	222 (50.6)	148 (62.4)	
G3	306 (45.3)	217 (49.4)	89 (37.6)	**0.004**
Clinical response to CT/RT				
Complete	253 (37.4)	153 (34.9)	100 (42.2)	
Partial	402 (59.5)	269 (61.3)	133 (56.1)	
Stable disease	21 (3.1)	17 (3.9)	4 (1.7)	0.072^c^

Bold values indicate statistically significant

^a^Calculated by Fisher’s exact test for proportions

^b^Mann–Whitney *U* test

^c^Calculated by Pearson’s *χ*^2^ test

*O-RS* open radical surgery, *MI-RS* minimally invasive radical surgery, *BMI* body mass index, *CT/RT* chemoradiotherapy

**Table 2:** Univariate and multivariate analysis of preoperative clinical and pathological features as prognostic factors for disease-free survival and disease-specific survival in the whole series

	Disease-free survival				Disease-specific survival			
	Univariate	*p* Value	Multivariate	*p* Value	Univariate	*p* Value	Multivariate	*p* Value
Age, years								
≤ 65	Ref.		Ref.		Ref.		Ref.	
> 65	0.801 (0.527–1.216)	0.297	0.814 (0.533–1.243)	0.341	0.885 (0.534–1.469)	0.637	0.789 (0.471–1.322)	0.368
FIGO stage								
IB2–IIB	Ref.		Ref.		Ref.		Ref.	
III–IVA	1.652 (1.155–2.363)	**0.006**	1.398 (0.960–2.301)	0.081	1.878 (1.221–2.888)	**0.004**	1.1527 (0.983–2.372)	0.059
Lymph node status at imaging								
Negative	Ref.		Ref.		Ref.		Ref.	
Pelvic positive	1.404 (1.024–1.924)	**0.035**	1.309 (0.950–1.803)	0.100	1.283 (0.862–1.909)	0.219	1.122 (0.750–1.678)	0.576
Aortic ± pelvic positive	3.947 (2.201–7.078)	< **0.001**	2.825 (1.554–5.138)	**0.001**	3.643 (1.802–7.367)	**< 0.001**	2.356 (1.149–4.830)	**0.019**
Histotype								
Squamous	Ref.		Ref.		Ref.		Ref.	
Other	1.223 (0.788–1.898)	0.370	1.070 (0.682–1.679)	0.767	1.184 (0.675–2.076)	0.555	1.075 (0.606–1.904)	0.804
Clinical response to CT/RT								
Complete	Ref.		Ref.		Ref.		Ref.	
Partial	2.352 (1.620–3.415)	< **0.001**	2.149 (1.471–3.141)	< **0.001**	3.327 (1.971–5.616)	**< 0.001**	3.005 (1.767–5.109)	< **0.001**
Stable disease	7.228 (3.885–13.448)	< **0.001**	6.053 (3.186–11.499)	< **0.001**	12.475 (5.830–26.695)	**< 0.001**	9.980 (4.543–21.921)	< **0.001**

Bold values indicate statistically significant

*CT/RT* chemoradiotherapy, *O-RS* open radical surgery, *MI-RS* minimally invasive radical surgery

PS matching resulted in a cohort of 462 patients (231 patients per group), well balanced in all variables (Table 3). We calculated that a sample of 462 patients would provide 90% power to declare MI-RS noninferior to O-RS in terms of DFS, on the basis of an expected DFS rate of 75% in patients managed by O-RS in our previous studies,^6^^,^^13^ with a noninferiority margin of − 6.4%. Supplementary Table 1 summarizes surgical details in the PS-weighted population. The frequency of type I–II RH was higher in the MI-RS group versus the O-RS group (*p* value < 0.001), while there was no difference between the two groups in terms of pelvic and aortic lymphadenectomy. A higher number of removed pelvic lymph nodes was documented in the O-RS groups versus the MI-RS group (*p* < 0.001). A total of 207 (44.8%) patients showed pCR (O-RS 45.0%, MI-RS 44.6%), 122 (26.4%) showed persistence of pmicroR (O-RS 27.3%, MI-RS 25.5%), and 133 (28.8%) presented pmacroR (O-RS 27.7%, MI-RS 29.9%) (Supplementary Table 2). Vaginal surgical margins were found positive in nine (1.9%) patients, with three (1.3%) cases in the O-RS group versus six (2.3%) in the MI-RS group (*p* = 0.313). Overall, adjuvant chemotherapy was only administered in 38 (16.4%) patients managed by O-RS and in 53 (22.9%) MI-RS patients (*p* = 0.101), vaginal brachytherapy ± adjuvant chemotherapy was administered in 4 (1.7%) patients in the O-RS group, and in 5 (2.2%) MI-RS patients (*p* = 0.571) (data not shown). Three patients were not administered adjuvant therapy because of poor clinical condition, while three patients refused any further treatment (data not shown).

**Table 3:** Preoperative clinical and pathological features in the PS-weighted population

	All (*N* = 462)	O-RS (*N* = 231)	MI-RS (*N* = 231)	*p* Value ^a^
Age, years				
Median (range)	52 (20–83)	53 (20–83)	51 (23–79)	0.125^b^
BMI, kg/m^2^				
Median, range	23.9 (16.4–45.0)	24.2 (17–36.5)	23.8 (16.4–45.0)	0.584^b^
FIGO stage				
IB2	40 (8.6)	20 (8.6)	20 (8.6)	
IIA	26 (5.6)	14 (6.1)	12 (5.2)	
IIB	342 (74.0)	170 (73.6)	172 (7.4)	
IIIA	17 (3.7)	9 (3.9)	8 (3.5)	
IIIB	37 (8.0)	18 (7.8)	19 (8.2)	0.993^c^
Tumor size				
≤ 4 cm	86 (18.6)	45 (19.5)	41 (17.7)	
> 4 cm	376 (81.4)	186 (80.5)	190 (82.2)	0.720
Lymph node status at imaging				
Negative	306 (66.2)	153 (66.2)	153 (66.2)	
Pelvic positive	145 (31.4)	73 (31.6)	72 (31.2)	
Aortic ± pelvic positive	11 (2.4)	5 (2.2)	6 (2.6)	0.952^c^
Histotype				
Squamous	406 (87.9)	207 (89.6)	199 (86.1)	
Other	56 (12.1)	24 (10.4)	32 (13.9)	0.493
Grade				
G1–G2	290 (62.8)	146 (63.2)	144 (62.3)	
G3	172 (37.2)	85 (36.8)	87 (37.7)	0.923
Clinical response to CT/RT				
Complete	195 (42.2)	96 (41.6)	99 (42.9)	
Partial	257 (55.6)	129 (55.8)	128 (55.4)	
Stable disease	10 (2.2)	6 (2.6)	4 (1.7)	0.798^c^

^a^Calculated by Fisher’s exact test for proportions

^b^Mann–Whitney *U* test

^c^Calculated by Pearson’s *χ*^2^ test

*O-RS* open radical surgery, *MI-RS* minimally invasive radical surgery

### Survival Analysis in PS-Weighted Population

**Fig. 2.**
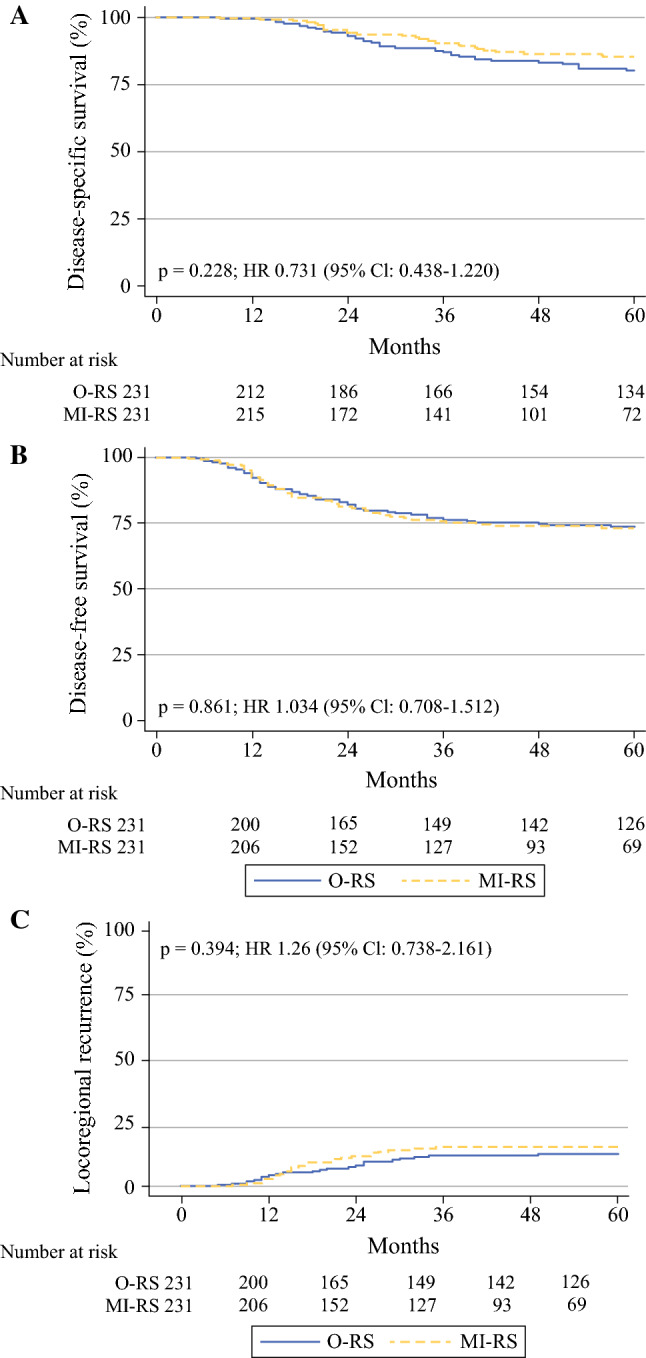
Cumulative curves for (**A**) disease-free survival (DFS) and (**B**) disease-specific survival (DSS); (**C**) cumulative incidence of locoregional recurrence in the PS-weighted population

As of November 2019, the median duration of follow-up was 43 months (range 4–118 months) in the MI-RS group, and 76 months (range 6–199 months) in the O-RS group. A total of 107 recurrences were registered, with no difference in the distribution of their pattern between the two surgical approaches (Supplementary Table 3). Figure 2 shows the Kaplan–Meier curves for DFS and DSS, and the cumulative incidence curve for locoregional recurrence. The 5-year DFS was 73.7% in the O-RS group versus 73.0% in the MI-RS group (HR 1.034, 95% CI 0.708–1.512, *p* = 0.861) (Fig. 2A). Death of disease was registered in 62 patients; the 5-year DSS was 80.4% in the O-RS group versus 85.3% in the MI-RS group (HR 0.731, 95% CI 0.438–1.220, *p* = 0.228) (Fig. 2B). The 5-year estimated locoregional recurrence rate was 12.5% in the O-RS group (HR 1.174, 95% CI 0.656–2.104, *p* = 0.588) versus 15.2% in the MI-RS group (Fig. 2C). As shown in Table 4, multivariate analysis of clinical outcome in the PS-weighted population showed that only clinical response to CT/RT maintained its independent impact on DFS and DSS. Supplementary Fig. 1 shows the Kaplan–Meier curves for DFS (A) and DSS (B) in patients presenting clinical complete response versus those achieving clinical partial response/stable disease.

**Table 4:** Univariate and multivariate analysis of preoperative clinical and pathological features, as well as and surgical approach as prognostic factors for disease-free survival and disease-specific survival in the PS-weighted population

	Disease-free survival				Disease-specific survival			
	Univariate	*p* Value	Multivariate	*p* Value	Univariate	*p* Value	Multivariate	*p* Value
Age, years								
≤ 65	Ref.		Ref.		Ref		Ref	
> 65	0.849 (0.505–1.425)	0.535	0.932 (0.553–1.571)	0.793	0.874 (0.444–1.719)	0.696	0.866 (0.437–1.717)	0.680
FIGO stage								
IB2–IIB	Ref.		Ref.		Ref.		Ref	
III–IVA	1.203 (0.697–2.077)	0.507	1.051 (0.601–1.838)	0.862	1.241 (0.612–2.517)	0.549	1.144 (0.557–2.349)	0.715
Lymph node status at imaging								
Negative	Ref.		Ref.		Ref.		Ref	
Pelvic positive	1.644 (1.108–2.440)	**0.013**	1.600 (1.073–2.384)	**0.021**	1.394 (0.822–2.363)	0.218	1.361 (0.797–2.322)	0.259
Aortic ± pelvic positive	2.758 (1.107–6.873)	**0.029**	2.097 (0.820–5.359)	0.122	2.182 (0.673–7.076)	0.194	1.367 (0.398–4.702)	0.620
Histotype								
Squamous	Ref.		Ref.		Ref.		Ref	
Other	0.974 (0.535–1.776)	0.933	0.834 (0.452–1.537)	0.560	1.008 (0.459–2.213)	0.985	0.893 (0.401–1.988)	0.781
Clinical response to CT/RT								
Complete	Ref.		Ref.		Ref.		Ref	
Partial	2.068 (1.343–3.183)	**0.001**	1.995 (1.287–3.092)	**0.002**	3.195 (1.692–6.033)	< **0.001**	3.137 (1.649–5.968)	< **0.001**
Stable disease	6.813 (2.819–16.464)	< **0.001**	6.619 (2.676–16.371)	< **0.001**	10.550 (3.400–32.737)	< **0.001**	10.430 (3.183–34.176)	< **0.001**
Surgical approach								
O-RS	Ref.		Ref.		Ref.		Ref	
MI-RS	1.034 (0.708–1.512)	0.861	1.029 (0.703–1.507)	0.883	0.731 (0.438–1.220)	0.231	0.720 (0.429–1.206)	0.212

Bold values indicate statistically significant

*CT/RT* chemoradiotherapy, *O-RS* open radical surgery, *MI-RS* minimally invasive radical surgery

### Perioperative Details and Intraoperative Complications

Supplementary Table 5 shows that operative time was longer in the MI-RS than O-RS group (*p* = 0.0351). Moreover, estimated blood loss was lower in the MI-RS versus O-RS group (*p* < 0.001). Length of hospital stay was shorter in the MI-RS versus O-RS group (*p* < 0.001). There were 19 (3.5%) intraoperative complications in the O-RS patients, and 11 (4.8%) in the MI-RS group (*p* = 0.492), of which 17 were successfully managed intraoperatively with no further consequences, while there were 2 severe intraoperative complications, namely one bowel injury requiring temporary ileostomy in the MI-RS group and one bladder lesion requiring ureteroneocystostomy in the O-RS group.

Overall, 22 cases (9.5%) required conversion from MI-RS to O-RS, due to fibrosis in most cases.

### Postoperative Complications

During the observation period, 165 (35.7%) patients in the PS-weighted population experienced early postoperative complications, for a total of 203 complications. The number of patients experiencing early postoperative complications was 77 (33.3%) in the O-RS group versus 88 (38.1%) in the MI-RS group (*p* = 0.331).

Late postoperative complications were registered in 117 patients, for a total of 141 complications. The number of patients experiencing late postoperative complications was 56 (24.2%) in the O-RS group versus 61 (26.4%) in the MI-RS group (*p* = 0.668) (Table 5).

**Table 5:** Distribution of number of patients experiencing postoperative complications in the PS-weighted population

	All (*N* = 462)	O-RS (*N* = 231)	MI-RS (*N* = 231)	*p* Value^a^
No. patients with early postoperative complications				
All	165 (35.7%)	77 (33.3%)	88 (38.1%)	0.331
Grade 1	105 (22.7%)	44 (19.4%)	61 (26.4%)	
Grade 2	46 (9.9%)	23 (9.9%)	23 (9.9%)	
Grade 3–4	14 (3.0%)	10 (4.3%)	4 (1.7%)	0.100
No. patients with late postoperative complications				
All	117 (25.3%)	56 (24.2%)	61 (26.4%)	0.668
Grade 1	52 (11.2%)	25 (10.8%)	27 (11.7%)	
Grade 2	54 (11.7%)	26 (11.2%)	28 (12.1%)	
Grade 3–4	11 (2.4%)	5 (2.2%)	6 (2.6%)	0.985

^a^Calculated by Pearson’s *χ*^2^ test

*O-RS* open radical surgery, *MI-RS* minimally invasive radical surgery

There was no difference in the distribution of severity of early or late postoperative complications between the two groups (Table 5).

Supplementary Table 5 and 6 summarize the distribution of early and late postoperative complications between the two groups: one patient in the O-RS group died within 30 days from surgery as a consequence of complicated abdominal abscess with subsequent peritonitis and multiorgan failure, and one patient in the MI-RS group died during adjuvant treatment due to drug-related toxicity.

## Discussion

Adoption of completion surgery as an alternative to vaginal brachytherapy after chemoradiation may play a role in LACC patients with suboptimal response to CT/RT or in low-income countries with limited access to radiotherapy.

To the best of the authors’ knowledge, this is the largest study evaluating the impact of MI-RS versus O-RS in a large, retrospective series of LACC patients managed by preoperative CT/RT and followed with long-term surveillance. The sample size resulting from the adoption of the propensity score approach was calculated to provide 90% power to declare MI-RS noninferior to O-RS in terms of DFS, considering the expected DFS rate of 75% in O-RS patients^6^ and a noninferiority margin of −6.4%.

Indeed, we showed that the MI-RS approach was noninferior to O-RS, with 5-year DFS of 73.0% in the MI-RS versus 73.7% in the O-RS group (HR 1.034, 95% CI 0.708–1.512, *p* = 0.861). Moreover, there was also no difference in terms of DSS between the two groups. These findings differ greatly from the results reported by the LACC trial,^28^ as well as by population-based observational studies in early-stage disease from the USA and other countries.^29^

Preoperative CT/RT is shown to provide pathologic complete or microscopic response in about 70% of patients in our series, a figure that matches well with other studies.^9^^,^^25^ Therefore, the very high rate of disease downstaging, including primary tumor and regional disease,^6^^,^^37^ facilitates the adoption of less-extensive radical surgery and the use of MI-RS, contributing to prevention of potential perioperative peritoneal contamination.^38^ Overall, the distribution of the patterns of relapse was not divergent between the two surgical approaches, and the cumulative incidence of locoregional relapse was not different between the MI-RS and O-RS groups.

It must be acknowledged that the study preplanned for the multivariate analysis to include only preoperative parameters (since the choice of surgical approach is based on the preoperative workup), with the aim of identifying variables with prognostic impact on DFS for the development of the PS-weighted sample. Therefore, one could argue that the exclusion of surgical and postoperative parameters from the PS matching could have biased the analysis due to the potential underestimation of additional prognostic factors, such as extent of surgery radicality, pathologically assessed response to CT/RT, or adjuvant treatment. As far as the patterns of pathologic response to CT/RT and adjuvant treatment are concerned, the distribution was not different between the two groups. On the other hand, patients managed by MI-RS underwent Piver I–II RH more frequently compared with those in the O-RS group (52.4% vs. 17.7%, respectively). Moreover, the number of pelvic lymph nodes removed was lower in the MI-RS patients compared with the O-RS patients. However, in spite of these findings, the clinical outcome was not worse in the MI-RS patients, thus reinforcing the concept that CT/RT results in a drastic reduction in the size of disease, thus allowing surgeons to safely adopt both minimally invasive approaches and less radical surgery according to the preoperatively assessed clinical response.

Some limitations of the study could have affected the survival analysis, such as the difference in duration of follow up between the two treatment groups and the relatively high rate of conversion (9.5%) from minimally invasive to open surgery, which in most cases was associated with technical difficulties rather than extent of disease.

It must be acknowledged that the adoption of MI-RS in our centers was approximately concomitant with the start of intensity-modulated radiotherapy techniques. These have been reported to deliver a lower dose to the small bowel and bladder than standard pelvic radiotherapy in CC patients, thus reducing acute and chronic adverse effects possibly related to inflammation and tissue fibrosis.^39^^,^^40^ Our findings of longer operative time, reduced EBL, and hospital stay in the MI-RS patients confirm previous data.^27^ Conversely, we could not confirm that MI-RS would provide a better toxicity profile compared with the open approach; indeed, the proportions of patients experiencing grade 2 or grade 3–4 postoperative morbidities were 9.9% and 3.0% for early complications, and 11.7% and 2.4% for late morbidities, respectively, without any statistically significant difference between the two surgical approaches. We do not exclude that, due to its retrospective design, our study could have been biased by the high risk of underreporting real morbidity, but it is conceivable that synergy between IMRT and adoption of MI-RS, as well as improvement of surgical and radiotherapy equipment, could have played a role.

In conclusion, we report that use of MI-RS in LACC patients does not impair clinical outcome compared with open surgery. Moreover, notwithstanding the limits inherent to the retrospective design of the study, the rate of postoperative complications was not different between the two groups. In this context, we might consider the adoption of minimally invasive surgery as the standard surgical approach to LACC patients after CT/RT in the future, especially in high-volume gynecological cancer units with surgeons experienced in laparoscopic and robotic gynecologic oncologic procedures. Based on the patient features/morbidities and the clinical response to CT/RT, surgeons could carefully tailor the extent of radical surgery, thus resulting in a personalized surgical approach for each patient. Moreover, the integration of multidisciplinary teams could help to prevent or attenuate postoperative morbidities, therefore preserving women’s quality of life.

Furthermore, the search for biomolecular parameters in cervical cancer tissue could help to identify a personalized signature capable of defining radiosensitivity and prognosis more successfully, as well as predisposition to radiotherapy toxicity and consequent surgery toxicity.

## Electronic supplementary material

Below is the link to the electronic supplementary material.

Supplementary material 1 (DOCX 15 kb)

Supplementary material 1 (DOCX 14 kb)

Supplementary material 1 (DOCX 15 kb)

Supplementary material 1 (DOCX 14 kb)

Supplementary material 1 (DOCX 18 kb)

Supplementary material 1 (DOCX 19 kb)

Supplementary material 1 (PPTX 126 kb)
