# Supplementary material for: Minimally Invasive Approaches in Locally Advanced Cervical Cancer Patients Undergoing Radical Surgery After Chemoradiotherapy: A Propensity Score Analysis
Source: Ann Surg Oncol. 2020 Nov 9;28(7):3616–26. doi: 10.1245/s10434-020-09302-y (PMC8184543; doi:10.1245/s10434-020-09302-y)
Supplement: Supplementary file 1 — Supplementary material 1 (DOCX 15 kb) [file 10434_2020_9302_MOESM1_ESM.docx]

**Supplementary Table 1. Surgical details in the PS-weighted population**

|  | **ALL**  **(N=462)** | **O-RS**  **(N=231)** | **MI-RS**  **(N=231)** | **p value**^a^ |
| --- | --- | --- | --- | --- |
| **Radical Hysterectomy**  I-II  III  IV | 162 (35.1)  280 (61.5)  16 (3.5) | 41 (17.7)  177 (76.6)  13 (5.6) | 121 (52.4)  107 (46.3)  3 (1.3) | **<0.001** |
| **Lymphadenectomy**  Pelvic only  Pelvic and aortic | 340 (73.6)  122 (26.4) | 171 (74.0)  60 (26.0) | 169 (73.2)  62 (26.8) | 0.916 |
| **N. pelvic lymph nodes removed**  Median (range) | 23 (2-73) | 25 (3-73) | 18 (2-57) | **<0.001**^b^ |
| **N. aortic lymph nodes removed**  Median (range) | 9 (1-54) | 8.5 (2-54) | 11 (1-46) | 0.353^b^ |

^a^calculated by Pearson’s χ2 test, ^b^Mann-Whitney U test
