# Supplementary material for: Minimally Invasive Approaches in Locally Advanced Cervical Cancer Patients Undergoing Radical Surgery After Chemoradiotherapy: A Propensity Score Analysis
Source: Ann Surg Oncol. 2020 Nov 9;28(7):3616–26. doi: 10.1245/s10434-020-09302-y (PMC8184543; doi:10.1245/s10434-020-09302-y)
Supplement: Supplementary file 2 — Supplementary material 1 (DOCX 14 kb) [file 10434_2020_9302_MOESM2_ESM.docx]

**Supplementary Table 2. Distribution of pathological response in the PS-weighted population**

|  | **ALL**  **(N=462)** | **O-RS**  **(N=231)** | **MI-RS**  **(N=231)** | **p value**^a^ |
| --- | --- | --- | --- | --- |
| **Pathologic response**  Complete  Microscopic  Macroscopic | 207 (44.8)  122 (26.4)  133 (28.8) | 104 (45.0)  63 (27.3)  64 (27.7) | 103 (44.6)  59 (25.5)  69 (29.9) | 0.850 |

^a^calculated by Pearson’s χ2 test
