# Supplementary material for: Minimally Invasive Approaches in Locally Advanced Cervical Cancer Patients Undergoing Radical Surgery After Chemoradiotherapy: A Propensity Score Analysis
Source: Ann Surg Oncol. 2020 Nov 9;28(7):3616–26. doi: 10.1245/s10434-020-09302-y (PMC8184543; doi:10.1245/s10434-020-09302-y)
Supplement: Supplementary file 3 — Supplementary material 1 (DOCX 15 kb) [file 10434_2020_9302_MOESM3_ESM.docx]

**Supplementary Table 3**. **Distribution and pattern of recurrences**

|  | **All**  **(N=462)** | **O-RS**  **(N=231)** | **MI-RS**  **(N=231)** | **p value**^a^ |
| --- | --- | --- | --- | --- |
| N. of recurrent patients (%) | 107 (23.2) | 54 (23.4) | 53 (22.9) | 0.306 |
| Site of recurrence, N. (%)^b^  -Pelvic central/lateral  -Aortic lymph nodes  -Parenchyma^c^  -Mixed | 33 (30.8)  8 (7.5)  32 (29.9)  34 (31.8) | 14 (25.9)  5 (9.2)  16 (29.6)  19 (35.2) | 19 (35.8)  3 (5.7)  16 (30.2)  15 (28.3) | 0.517 |

^a^calculated by Pearson's χ2 test; ^b^calculated on the recurrent patients; ^c^Lung, liver and brain
