# Supplementary material for: Minimally Invasive Approaches in Locally Advanced Cervical Cancer Patients Undergoing Radical Surgery After Chemoradiotherapy: A Propensity Score Analysis
Source: Ann Surg Oncol. 2020 Nov 9;28(7):3616–26. doi: 10.1245/s10434-020-09302-y (PMC8184543; doi:10.1245/s10434-020-09302-y)
Supplement: Supplementary file 4 — Supplementary material 1 (DOCX 14 kb) [file 10434_2020_9302_MOESM4_ESM.docx]

**Supplementary Table 4. Peri-operative details and intra-operative complications in the**

**PS-weighted population**

|  | **O-RS**  **(N=231)** | **MI-RS**  **(N=231)** | **p value**^a^ |
| --- | --- | --- | --- |
| **Operative time,** min  Median (range) | 231.5 (120-464) | 250 (65-600) | **0.0351** |
| **Estimated blood loss,** cc  Median (range) | 250 (50-1,500) | 100 (10-900) | **<0.001** |
| **Lenght of hospitalization,** days  Median (range) | 8 (3-42) | 4 (2-60) | **<0.001** |
|  |  |  |  |
| **N. patients with intra-operative**  **complications** | 8 (3.5%) | 11 (4.8%) | 0.492^b^ |
| -Bladder injury  -Bowel injury  -Vascular injury  -Ureteral injury | 3  -  4  1 | 4  2  3  2 |  |
| **Conversion to O-RS** | 22 (9.5%) | - | **-** |

^a^Mann-Whitney U test, ^b^calculated by Fisher’s exact test for proportions,

O-RS: open radical surgery, MI-RS: minimally invasive radical surgery
