# Supplementary material for: Minimally Invasive Approaches in Locally Advanced Cervical Cancer Patients Undergoing Radical Surgery After Chemoradiotherapy: A Propensity Score Analysis
Source: Ann Surg Oncol. 2020 Nov 9;28(7):3616–26. doi: 10.1245/s10434-020-09302-y (PMC8184543; doi:10.1245/s10434-020-09302-y)
Supplement: Supplementary file 5 — Supplementary material 1 (DOCX 18 kb) [file 10434_2020_9302_MOESM5_ESM.docx]

|  | **O-RS**  **N=231** |  | **MI-RS**  **N=231** | **p value** |
| --- | --- | --- | --- | --- |
| **N. patients with early**  **postoperative complications** | **77** **(33.3%)** |  | **88** **(38.1%)** | 0.332 |
| **N. complications** | **100** |  | **103** |  |
| ***Urinary*** | **31** | ***Urinary*** | **28** |  |
| *G1*  *-Occasional urinary incontinence*  *-Abnormal bladder function for <6 mts*  *-Urinary retention for <6 months* | *4*  *1*  *4* | *G1*  *-Occasional urinary incontinence*  *-Urinary retention for <6 months*  *- Abnormal bladder function for <6 mts* | *5*  *3*  *4* |  |
| *G2*  *-Postural incontinence*  *-Ureteral stenosis requiring surgery*  *with normal renal function*  *-Urinary fistula requiring surgery* | *5*  *6*  *3* | *G2*  *-Postural incontinence*  *-Ureteral stenosis requiring surgery*  *with normal renal function*  *-Urinary fistula requiring surgery* | *7*  *3*  *2* |  |
| *G3*  *-Urinary fistula with inadequate*  *renal function or permanent*  *nephrostomy*  *-Total incontinence*  *-Ureteral stenosis with inadequate*  *renal function or permanent*  *nephrostomy*  *-Permanent urinary retention*  *requiring long term catheter* | *2*  *1*  *2*  *3* | *G3*  *-Urinary fistula with inadequate*  *renal function or permanent*  *nephrostomy* | *4* |  |
| ***Vascular*** | **47** | ***Vascular*** | **53** |  |
| G1  -Leg edema  -Lymphocele  -Venous thromboembolism | 28  12  2 | G1  -Leg edema  -Lymphocele  - Venous thromboembolism | 34  10  1 |  |
| G2  -Intermittent or permanent leg edema  interfering with normal activity  -Lymphocele reguiring drainage  -Pulmonary embolism | 2  2  1 | G2  -Intermittent or permanent leg edema interfering with normal activity  -Lymphocele reguiring drainage | 6  2 |  |
| ***Gastro-intestinal*** | **6** | ***Gastro-intestinal*** | **5** |  |
| G1  -Obstruction not requiring surgery  -Proctitis | 3  1 | G1  -Obstruction not requiring surgery  -Proctitis | 2  2 |  |
| G2  -Small bowel obstruction requiring  surgery with normal function | 1 | G2  -Small bowel obstruction requiring  surgery with normal function | 1 |  |
| G3  -Sigmoid obstruction requiring surgery | 1 | G3  - | - |  |
| ***Other*** | **16** | ***Other*** | **17** |  |
| *G1*  *-Wound infection*  *-Neurological sensory symptoms* | *3*  *4* | *G1*  *-Pelvic abscess* | *9* |  |
| *G2*  *-Wound infection*  *-Wound dehiscence or laparocele*  *requiring surgery* | *4*  *3* | *G2*  *-Pelvic abscess requiring surgery*  *-Vaginal cuff dehiscence*  *- Incisional Hernia* | *5*  *2*  *1* |  |
| *G3*  *-Peritonitis requiring laparotomy* | *1* | *G3*  *-* | *-* |  |
| *G4*  *-Peritonitis, multi-organ failure* | *1* | *G4*  *-* | *-* |  |

**Supplementary Table 5. Early postoperative complications in the PS-weighted population**

^a^calculated by Fisher’s exact test for proportions, MI-RS: minimally invasive radical surgery, O-RS: open radical surgery
