# Supplementary material for: Minimally Invasive Approaches in Locally Advanced Cervical Cancer Patients Undergoing Radical Surgery After Chemoradiotherapy: A Propensity Score Analysis
Source: Ann Surg Oncol. 2020 Nov 9;28(7):3616–26. doi: 10.1245/s10434-020-09302-y (PMC8184543; doi:10.1245/s10434-020-09302-y)
Supplement: Supplementary file 6 — Supplementary material 1 (DOCX 19 kb) [file 10434_2020_9302_MOESM6_ESM.docx]

**Supplementary Table 6. Late post-operative complications in the PS-weighted population**

|  | **O-RS**  **N=231** |  | **MI-RS**  **N=231** | **p value** |
| --- | --- | --- | --- | --- |
| **N. patients with late**  **postoperative complications** | **56 (24.2%)** |  | **61 (26.4%)** | 0.669 |
| **N. complications** | **65** |  | **76** |  |
| ***Urinary*** | **20** |  | **24** |  |
| *G1*  *-Occasional urinary incontinence*  *-Abnormal bladder function*  *for <6 mts* | 3  2 | *G1*  *-Occasional urinary incontinence*  *-Urinary retention for < 6months*  *-Abnormal bladder function for <6 mts* | 4  2 |  |
| *G2*  *-Postural incontinence*  *-Ureteral stenosis requiring surgery*  *with normal renal function*  *-Urinary fistula requiring surgery*  *-Urinary retention requiring temporary catheter*  *- Abnormal bladder function for >6 mts* | 1  3  1  2  5 | *G2*  *-Postural incontinence*  *-Ureteral stenosis requiring surgery*  *with normal renal function*  *-Urinary fistula requiring surgery*  *-Urinary retention requiring temporary catheter*  *- Abnormal bladder function for >6 mts* | 3  8  1  3 |  |
| *G3*  *- Ureteral stenosis with inadequate*  *renal function or permanent*  *nephrostomy*  *-Total incontinence*  *-Permanent urinary retention requiring long term catheter* | 1  1  1 | *G3*  *-Ureteral stenosis with inadequate*  *renal function or permanent*  *nephrostomy*  *-Total incontinence*  *-Permanent urinary retention requiring long term catheter* | 1  1  1 |  |
| **Vascular** | **29** | ***Vascular*** | **40** |  |
| *G1*  *-Leg edema* | 20 | *G1*  *-Leg edema*  *-Lymphocele* | 26  1 |  |
| *G2*  *-Intermittent or permanent leg edema interfering with normal activity*  *-Lymphocele reguiring drainage* | 8  1 | *G2*  *-Intermittent or permanent leg edema interfering with normal activity*  *-Lymphocele reguiring drainage* | 10  3 |  |
| **Gastro-intestinal** | **4** | ***Gastro-intestinal*** | **2** |  |
| *G1*  *-Proctitis* | 1 | *G1*  *-* | - |  |
| *G2*  *-Small bowel obstruction requiring*  *surgery with normal function* | 1 | *G2*  *-* | **-** |  |
| *G3*  *-Bowel perforation* | 1 | *G3*  *-Colonic obstruction requiring surgery* | 2 |  |
| **Other** | **12** | ***Other*** | **10** |  |
| *G1*  *-Neurological sensory symptoms*  *-Vaginal cuff dehiscence* | 5  1 | *G1*  *-Neurological sensory symptoms* | 4 |  |
| *G2*  -L*aparocele requiring surgery*  *-Moderate dyspaurenia* | 4  1 | *G2*  *-Vaginal cuff dehiscence*  *-Moderate dyspareunia* | 4  1 |  |
| *G3*  *-Severe dyspareunia* | 1 | *G3*  *-* | - |  |
| *G4*  *-* | - | *G4*  *-Hematological toxicity* | 1 |  |

^a^calculated by Fisher’s exact test for proportions

O-RS: open radical surgery, MI-RS: minimally invasive radical surgery
