# Supplementary figures and images for: Minimally Invasive Approaches in Locally Advanced Cervical Cancer Patients Undergoing Radical Surgery After Chemoradiotherapy: A Propensity Score Analysis
Source: Ann Surg Oncol. 2020 Nov 9;28(7):3616–26. doi: 10.1245/s10434-020-09302-y (PMC8184543; doi:10.1245/s10434-020-09302-y)

## Slide 1
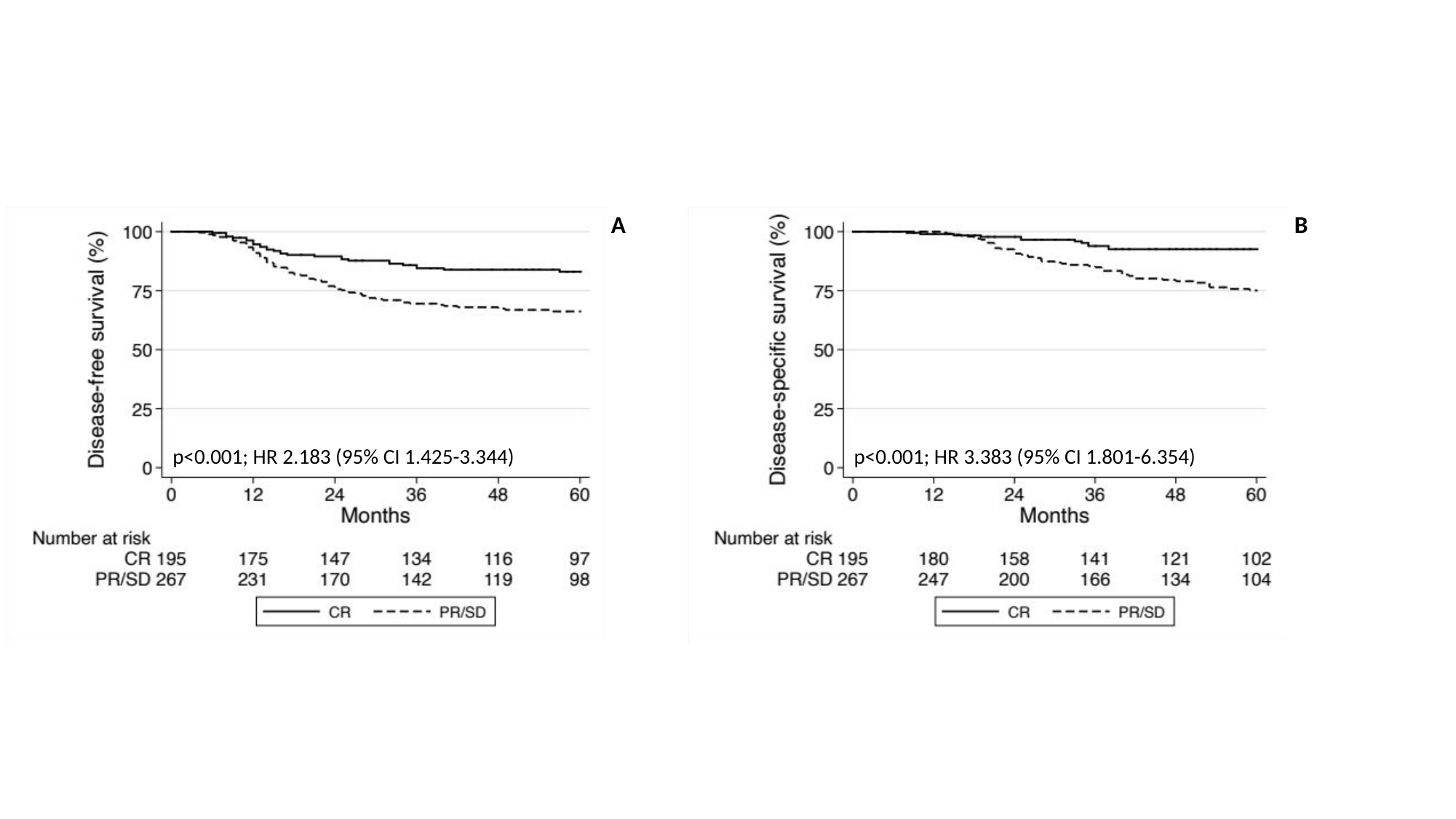

A
B
p<0.001; HR 2.183 (95% CI 1.425-3.344)
p<0.001; HR 3.383 (95% CI 1.801-6.354)

Supplement: Supplementary file 7 — Supplementary material 1 (PPTX 126 kb) [file 10434_2020_9302_MOESM7_ESM.pptx]
